# Impact of Deep-Learning-Based Respiratory Motion Correction on [^18^F] FDG PET/CT Test–Retest Reliability and Consistency of Tumor Quantification in Patients with Lung Cancer

**DOI:** 10.3390/biomedicines14010245

**Published:** 2026-01-21

**Authors:** Shijia Weng, Limei Jiang, Runze Wu, Yuanyan Cao, Yuan Li, Qian Wang

**Affiliations:** 1 Department of Nuclear Medicine, Peking University People’s Hospital, No.11 Xizhimen South Street, Xicheng District, Beijing 100044, China; 2 Beijing United Imaging Research Institute of Intelligent Imaging, Beijing 100094, China

**Keywords:** PET, respiratory motion correction, lung cancer, test–retest reliability, image quality

## Abstract

**Objectives:** Respiratory motion degrades the quantitative accuracy and test–retest (TRT) reliability of fluorine-18 fluorodeoxyglucose ([^18^F] FDG) positron emission tomography (PET)/computed tomography (CT) in lung cancer. This study investigated whether a deep-learning-based respiratory motion correction (RMC) method improves the TRT reliability and image quality of [^18^F] FDG PET tumor quantification compared with non-motion-corrected (NMC) reconstructions. **Methods:** Thirty-one patients with primary lung cancer underwent three PET acquisitions: whole body free breathing (Scan1), thoracic free breathing (Scan2), and thoracic controlled breathing (ScanCB). Each dataset was reconstructed with and without RMC. Visual assessments of liver motion artifacts, lesion clarity, and PET-CT co-registration were scored. Lung tumors were segmented to derive standardized uptake value max (SUVmax), SUVmean, metabolic tumor volume (MTV), PET-derived lesion length (PLL), and total lesion glycolysis (TLG). Visual image scores and TRT reliability of tumor quantification were compared using Kruskal–Wallis one-way analysis of variance and intraclass correlation coefficients (ICCs). **Results:** RMC reconstructions achieved higher visual scores of lesion clarity and PET-CT co-registration across all lung lobes and significantly reduced liver motion artifacts compared with NMC reconstructions. Differences in SUVmax, SUVmean, PLL, MTV, and TLG between Scan2 and ScanCB were significantly smaller with RMC than with NMC. ICCs for SUVmax, SUVmean, MTV, and TLG were higher between scans with RMC than NMC reconstructions, indicating improved TRT reliability. **Conclusions:** The deep-learning-based RMC method improved the image quality and TRT reproducibility of [^18^F] FDG PET/CT quantification in lung cancer, supporting its potential for routine adoption in therapy-response assessments.

## 1. Introduction

Positron emission tomography/computed tomography (PET/CT) integrates metabolic and anatomical information, substantially improving the diagnosis, staging, restaging, and treatment monitoring of lung cancer [1,2,3]. However, respiratory motion remains a major source of image degradation in lung cancer PET/CT imaging. It can cause lesion blurring, inaccurate attenuation correction, and misregistration between PET and CT images, leading to localization errors on the fused image [4,5,6]. Moreover, lung cancer patients with impaired pulmonary function may have difficulty maintaining regular breathing during PET/CT examinations, while treatment-induced changes in respiratory status, whether improvement or deterioration, can further compromise the image quality of PET/CT. Small isolated lung lesions are particularly vulnerable to respiratory artifacts, making accurate delineation, staging, and localization challenging in clinical practice [7,8]. Therefore, robust test–retest reliability in sequential PET/CT imaging is important for ensuring reliable longitudinal interpretation and consistent quantitative tumor assessment.

Several strategies have been proposed to mitigate respiratory motion in PET/CT imaging, including prone positioning, breath-hold protocols, device-assisted gating, and a data-driven respiratory gating technique [9,10,11,12]. However, each approach carries practical limitations. Gated acquisitions increase scan duration, require additional external equipment, or depend on reconstruction algorithms that reduce workflow speed. Breath-holding or prone position scanning are often constrained by patient compliance and variable respiratory capacity.

A recently developed deep-learning-based solution (uExcel OncoFocus, United Imaging Healthcare, Shanghai, China) addresses these challenges by integrating respiratory motion correction (RMC) directly into the reconstruction process. Briefly, respiratory signals are first extracted directly from PET list-mode data using a centroid-of-distribution (COD) approach constrained to an anatomically defined thoracoabdominal region, which is segmented by a deep-learning–based cavity segmentation network. The PET data are subsequently sorted into respiratory gates. A second deep-learning network then predicts gated attenuation maps from non-attenuation-corrected PET images, thereby enabling phase-matched attenuation correction for each respiratory gate. The gated PET images are non-rigidly registered to a reference respiratory phase and combined to correct inter-frame motion blur while preserving the full set of acquired PET counts. To address the long-standing issue of PET–CT misalignment, OncoFocus further performs non-rigid registration between the deep-learning–generated attenuation map of the reference respiratory gate and the CT-derived attenuation map. The resulting deformation field is subsequently applied to the motion-corrected PET image, effectively reformulating the PET–CT alignment problem as a single-modality registration task. Previous studies have shown that RMC reconstructions enhance lesion conspicuity, improve PET/CT co-registration, and recover previously occult lesions without additional hardware or prolonged acquisition [13].

Nevertheless, the influence of RMC reconstruction on scan–to-scan variability and quantitative consistency has not been comprehensively evaluated. Therefore, in this study, we conducted a test–retest (TRT) analysis to investigate whether the RMC algorithm improves the TRT reliability and image quality of [^18^F]FDG PET/CT tumor quantification in patients with lung tumors compared with non-motion-corrected (NMC) reconstructions.

## 2. Methods

### 2.1. Patient Characteristics

A total of 31 patients with lung cancer were prospectively enrolled at our center between 1 January 2025 and 31 January 2025. Histopathological subtypes included 12 adenocarcinomas, 11 squamous cell carcinomas, and 8 other variants of small cell lung cancer. All patients were clinically staged as cI to cII, with a maximal diameter <5 cm. All participants successfully underwent [^18^F] FDG PET/CT examinations. There were 12 females and 19 males with a mean age of 57.8 ± 12.1 years (range: 31–75 years). Seventeen tumors were located in the middle-upper lobe (MU-Lobe group) and 14 in the lower lobe (L-Lobe group). This study was reviewed and approved by our Institutional Ethics Committee, and all methods adhered to the ethical principles of the Declaration of Helsinki. All participants signed written informed consent.

### 2.2. PET/CT Imaging Acquisitions

All patients fasted for at least 6 h prior to the examination, and blood glucose levels on the day of scanning were below 11.1 mmol/L. [^18^F] FDG (Beijing Atomic High-tech Co., Ltd., Beijing, China) was intravenously administered at a dose of 4.07~4.44 MBq per kilogram of body weight. After an uptake period of about 50 min, PET/CT was performed using a United Imaging uMI Panorama 35S scanner (United Imaging Healthcare, Shanghai, China). PET data were acquired in 3D mode with 2.5 min per bed position. CT scanning was performed with 120 kVp tube voltage and automatic tube current modulation. PET images were reconstructed using a slice thickness of 2.8 mm and the corresponding CT images were used for attenuation correction and PET/CT image fusion. During PET data acquisition, patients were positioned with their arms alongside their body to minimize motions.

**Figure 1:**
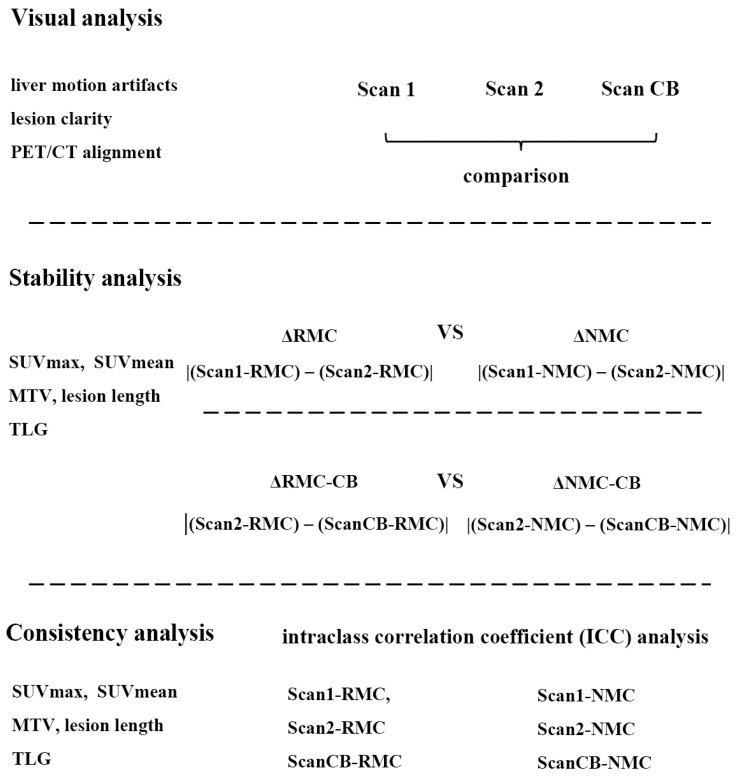
Flowchart of the study design. CB: controlled breathing; MTV: metabolic tumor volume; and TLG: total lesion glycolysis.

All patients underwent three PET acquisitions (Figure 1): a multi-bed whole body PET scan from the skull base to the mid-thigh during free breathing (Scan1), a single-bed PET scan at the thoracic region during free breathing for 2.5 min (Scan2), and a thoracic PET scan with controlled breathing (ScanCB) that includes 20 s initial breath-holding followed by free breathing for the remaining 2.5 min. The PET datasets were reconstructed with and without respiratory motion correction (RMC and NMC reconstruction, respectively) for the three scans, which produce six reconstruction sets: RMC-1, RMC-2, RMC-CB, NMC-1, NMC-2, and NMC-CB, corresponding to Scan1, Scan2, and ScanCB, respectively.

### 2.3. Data Analyses

**Figure 2:**
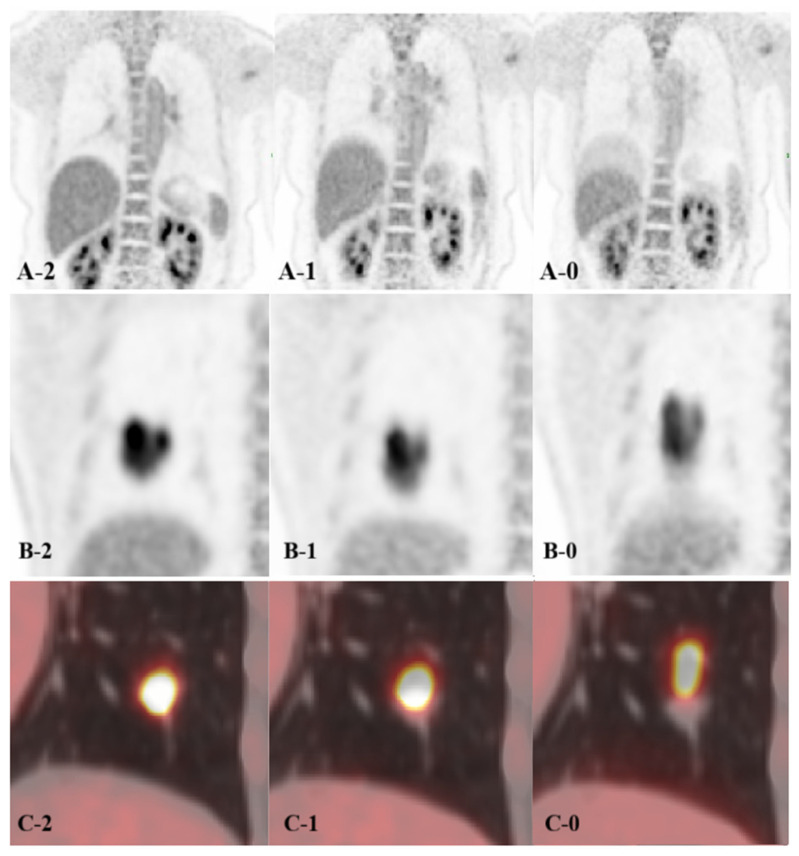
Visual assessment of PET/CT imaging. **A**: The respiratory motion of the liver; **B**: lesion clarity; and **C**: PET/CT alignment. The visual scores: good (**2**), moderate (**1**), and poor (**0**).

Subjective assessments of motion artifacts, image quality, and PET/CT alignment were performed independently by two experienced nuclear medicine physicians using a 3-point Likert scale. Both readers were blinded to the reconstruction method. If there were discrepancies, the consensus scores were determined through a joint review with a third senior radiologist. The following criteria were used (Figure 2). Liver motion artifacts were assessed by measuring the vertical distance between the highest point of the artifact and the liver on coronal PET images (0: severe artifacts with distance >5 cm; 1: mild artifacts, <5 cm; and 2: no visible artifacts); lesion clarity was rated according to lesion distortion and impacts on diagnostic interpretation (0: severe deformation or double-edge lesion images that may affect diagnosis confidence; 1: mild deformation with minimal diagnostic impact; and 2: no visible deformation); and PET/CT alignment was assessed on fusion images according to the degree of spatial overlap between PET and CT for lesions (0: poor alignment with no overlap; 1: moderate alignment with at least partial overlap; and 2: good alignment without visible mismatch).

Quantitative evaluation on lung tumors was performed by a designated nuclear medicine physician using a semi-automatic contouring tool on a commercial image processing workstation. Three-dimensional volumes of interest (VOIs) were delineated for each lesion using a 40% SUVmax threshold. The following quantitative parameters were extracted: maximum standardized uptake value (SUVmax), mean SUV (SUVmean), metabolic tumor volume (MTV), PET-derived lesion length (PLL), and total lesion glycolysis (TLG).

To evaluate TRT reliability, differences in quantitative parameters between Scan1 and Scan2 were calculated for both RMC and NMC reconstructions, denoted as ΔRMC and ΔNMC, respectively. These were defined as ΔRMC = |(RMC of Scan1) − (RMC of Scan2)| and ΔNMC = |(NMC of Scan1) − (NMC of Scan2)|, where RMC and NMC represent the parameter values obtained from RMC and NMC reconstructions. This computation was applied individually to SUVmax, SUVmean, MTV, PLL, and TLG. Similarly, comparisons between Scan2 and ScanCB were performed and denoted as ΔRMC-CB and ΔNMC-CB. In addition, the coefficients of variation (CVs) of all tumor quantification parameters across the three scans were calculated and compared between RMC and NMC reconstructions to assess quantitative stability.

To disentangle the effects of respiratory motion amplitude and the efficacy of RMC, subgroup analyses were performed based on lesion location in middle-upper or lower lung lobes for both visual and quantitative analysis, where applicable. A schematic overview of the data analysis workflow is presented in Figure 1.

### 2.4. Statistical Analysis

All statistical analyses were performed using SPSS software (version 27.0). The Kruskal–Wallis one-way analysis of variance by ranks was used to compare categorical scores from the visual assessments. Paired *t*-tests were applied to analyze the differences in quantitative tumor parameters between RMC and NMC reconstructions. The intraclass correlation coefficients (ICCs) were calculated to assess the scan-to-scan consistency [14] and test–retest reliability of quantitative parameters between the RMC and NMC reconstructions. A *p*-value < 0.05 was considered statistically significant.

## 3. Results

### 3.1. Visual Analysis

**Figure 3:**
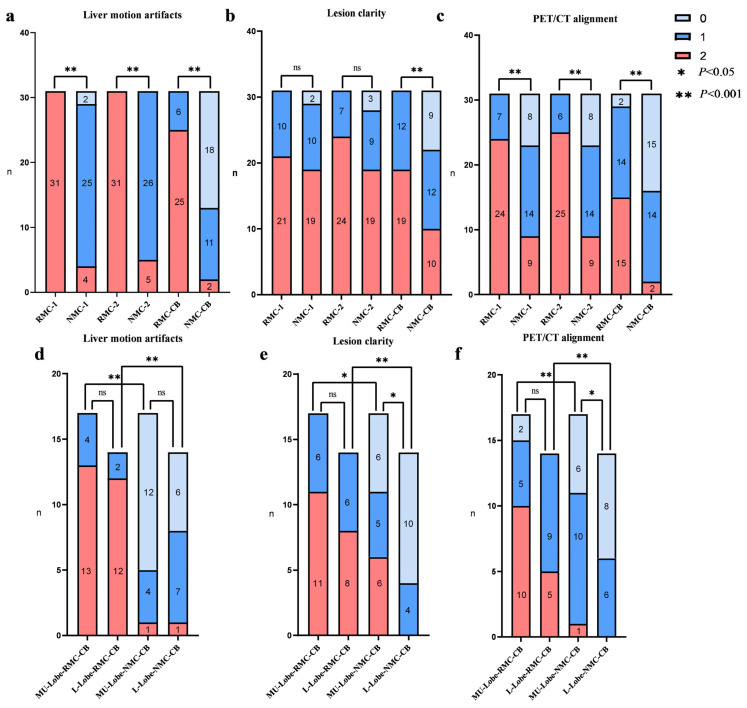
Scores of respiratory motion of the liver (**a**), lesion clarity (**b**), and PET/CT alignment for PET/CT imaging (**c**) with and without respiratory motion correction for three scans, and subgroup analysis is performed on ScanCB (**d**–**f**). MU-Lobe: middle-upper lobe; L-Lobe: lower lobe.

In the NMC reconstructions, visual scores for liver motion artifacts, lesion clarity, and PET/CT alignment in Scan 1 and Scan 2 were significantly better than those in ScanCB (*p* < 0.05). In contrast, no statistically significant differences were observed among three acquisitions with RMC reconstruction (*p* > 0.1). Across all scans, RMC reconstructions demonstrated significantly reduced liver motion artifacts and improved PET/CT alignment compared with NMC reconstructions (*p* < 0.05) (Figure 3a,c). Although overall lesion clarity scores did not differ significantly between RMC and NMC reconstructions in Scan1 and Scan2, superior lesion clarity was found in ScanCB (*p* < 0.005) (Figure 3b).

**Figure 4:**
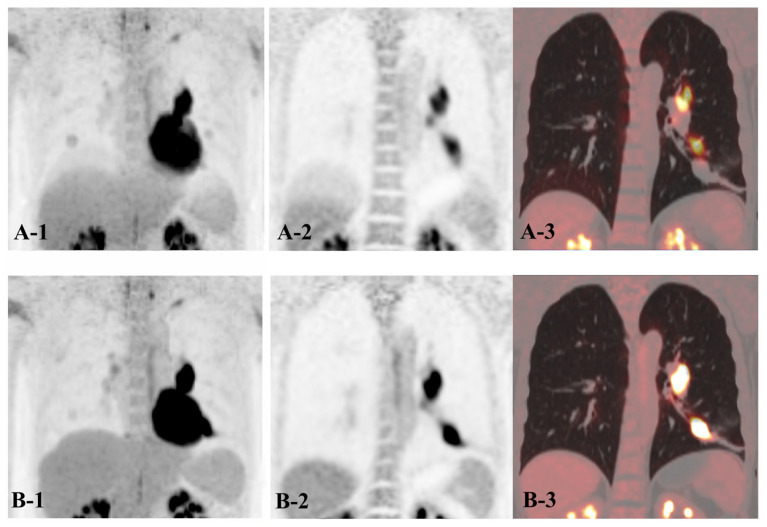
The scan CB image of a 47-year-old patient diagnosed with small cell lung cancer in the left lower lobe accompanied by left hilar lymph node metastasis. **A** denotes NMC reconstruction (**A-1**: PET Maximum intensity projection, **A-2**: PET coronal view, and **A-3**: PET/CT fusion image); the visual assessment yields poor or moderate scores on respiratory motion of the liver (0 point), PET/CT alignment (1 point), and lesion clarity (1 point). **B** denotes RMC reconstruction (**B-1**: PET Maximum intensity projection, **B-2**: PET coronal view, and **B-3**: PET/CT fusion image); the visual assessment yields good scores on respiratory motion of the liver (2 points), PET/CT alignment (2 points), and lesion clarity (2 points).

Subgroup analysis of ScanCB with NMC reconstruction showed that, except for liver motion artifacts (*p* > 0.1), both lesion clarity and PET/CT alignment were significantly better in the MU-Lobe group than in the L-Lobe group (both *p* < 0.05). In contrast, no significant inter-lobar differences were observed in the RMC reconstructions (*p* > 0.1). Furthermore, RMC reconstructions provided consistently better scores for liver artifacts, lesion clarity, and PET/CT alignment across all lobe groups compared with NMC reconstructions (all *p* < 0.05; Figure 3d–f). One characteristic imaging is shown in Figure 4.

### 3.2. Stability Analysis

In the comparison between Scan1 and Scan2 (Table 1), the mean difference in SUVmax of all lesions decreased from 0.88 ± 0.84 with NMC reconstructions to 0.57 ± 0.77 with RMC reconstructions (*p* < 0.05). In the lower-lobe subgroup, differences in TLG were also significantly smaller with RMC than NMC reconstruction (*p* < 0.1). No significant differences were observed in SUVmean in either the overall or subgroup analyses (*p* > 0.1).

In the comparison between Scan2 and ScanCB (Table 1), differences in SUVmax, SUVmean, PLL, MTV, and TLG were all significantly smaller with RMC reconstructions than NMC reconstructions (*p* < 0.05) in both the overall and subgroup analyses.

The CVs across the three scans are summarized in Table 2. With RMC reconstruction, CV values for all quantitative metabolic parameters (SUVmax, SUVmean, MTV, and TLG) and PLL were significantly lower than those with NMC reconstruction (*p* < 0.05) in both the overall and subgroup analyses. In the NMC reconstructions, the lower lobe lesions exhibited higher CV values compared with those in the middle-upper lobes. However, with RMC reconstruction, no significant inter-lobar differences were found, indicating reduced motion-related variablity (*p* > 0.1).

### 3.3. Consistency Analysis

The results of consistency analysis for quantitative metabolic parameters obtained from RMC and NMC reconstructions are listed in Table 3. For the NMC reconstructions, MTV and TLG showed excellent consistency across all lesions and subgroup analyses (all ICCs > 0.90). In contrast, SUVmean had relatively lower consistency in L-Lobe group (ICC = 0.729) compared with the overall and MU-Lobe group (all ICCs > 0.90). The PLL showed good consistency across all lesions and subgroup analyses (0.60 < ICCs < 0.90). SUVmax exhibited moderate consistency in the overall analysis (ICC = 0.505) and poor consistency in the L-Lobe group (ICC = 0.196).

With RMC reconstruction, all quantitative parameters (SUVmax, SUVmean, MTV, TLG) demonstrated excellent agreement (ICC > 0.90) in overall and subgroup analyses. All ICC values were higher with RMC than with NMC reconstruction, indicating improved scan-to-scan reliability and reduced respiratory motion–related variability.

## 4. Discussion

In this study, we evaluated the impact of a deep-learning-based RMC on the TRT stability and consistency of [^18^F] FDG PET/CT tumor quantification in patients with primary early-stage lung cancer. Our findings demonstrate that RMC reconstruction reduced respiratory motion artifacts, and therefore improved lesion clarity and PET-CT co-registration compared with NMC reconstruction. Furthermore, RMC reconstruction enhanced TRT reliability and tumor quantification consistency across repeated scans, which support its usage in quantitative and longitude oncologic PET/CT studies.

Respiratory motion is a major cause of image artifacts in thoracoabdominal PET imaging. It can degrade image quality through the loss of quantitative precision, misalignment between PET and CT images, and attenuation correction errors, leading to inaccurate lesion localization and metabolic assessment [15,16]. With the advances in imaging technology, deep learning-based techniques have gained widespread adoption in PET imaging and reconstruction [17,18,19]. Recently, a unified deep learning-based respiratory motion correction framework (uExcel OncoFocus) was introduced to address motion-induced artifacts without requiring external gating devices or extended acquisition time. In a previous study, RMC was shown to markedly improve the accuracy of SUV and volume quantification in lesions by effectively reducing respiratory motion-induced artifacts [20]. In this study, we systematically investigated the stability and consistency of this RMC approach and demonstrated its robust performance in improving both the visual and quantitative aspects of PET/CT imaging in lung cancer.

RMC was shown to markedly improve the accuracy of standardized uptake value (SUV) and volume quantification in lesions by effectively reducing respiratory motion-induced artifacts. These findings are consistent with prior studies that have reported comparable benefits of elastic respiratory motion correction techniques across diverse imaging applications [13,20].

Visual assessment demonstrated that NMC reconstructions had more pronounced motion artifacts in the liver, less lesion clarity, and poorer PET/CT alignment across all three acquisitions. In contrast, RMC constructions had fewer liver motion artifacts, improved lesion delineation, and more accurate PET-CT co-registration. These improvements were consistent across all scan groups, which indicates that RMC is robust to the respiratory heterogeneity inherent in both single-bed and overlapped multi-bed acquisitions. As a result, RMC reconstructions delivered reproducible, high-quality images independent of patient respiratory compliance. Furthermore, the RMC group had better visual image quality scores than the NMC group in all scans. By retaining the full PET photon count, the RMC algorithm effectively suppresses respiratory motion artifacts without compromising image quality, which is consistent with previous research [13].

In PET imaging, high TRT reliability and consistency of quantitative analysis is particularly important for tumor diagnosis and the precise assessment of tumor response to treatment [21,22]. Between scan1 and scan2, the RMC group had relatively smaller differences than the NMC group in SUVmax, PLL, and MTV. Similarly, between Scan1 and ScanCB, the average differences were relatively smaller in the RMC group than the NMC group. These differences were more pronounced in PLL and MTV, suggesting superior reliability and consistency of lesion morphological and volumetric measurements with RMC.

CV is often used as a primary metric of stability [23]. A previous study reported that, under a strict imaging protocol, the within-subject CVs could reach 10% and 11% for SUVmean and SUVmax, respectively. In our study, the CVs for SUVmean and SUVmax were lower than 10% with RMC reconstruction and were significantly lower than those in the NMC group. These results further confirm that RMC provides improved TRT reliability and quantitative robustness.

In PET imaging, quantitative consistency is fundamental to the validity of any clinical or research endpoints and underpins the reliable assessment of disease status, diagnostic reproducibility, and confidence [24,25]. Our study is the first to use the TRT method to explore the reliability and reproducibility of RMC in early lung cancer lesions. In the present study, SUVmax derived from NMC reconstructions only achieved moderate test–retest agreement (ICC = 0.505) when all lesions were considered, and dropped to poor agreement for lesions in the lower lung lobes (ICC = 0.196). This decline is likely attributable to the greater influence of respiratory motion in the lower lobes, where diaphragmatic displacement is most pronounced. In contrast, all quantitative parameters obtained from RMC reconstructions, including SUVmax, SUVmean, MTV, and TLG, demonstrated excellent TRT consistency across all lesions and subgroups. These findings confirm the superiority of motion-corrected quantification in improving the reproducibility and robustness of PET-derived metrics. The persistent reduction in the inaccuracy of early lung cancer lesion localization related to respiratory motion highlights the reliability of RMC as a valuable tool, which can improve diagnostic accuracy, particularly in scenarios where respiratory motion may compromise image quality and quantitative measurements. In contrast to conventional strategies, including breath-hold protocols, device-based or data-driven gating, standard registration-based motion correction, or deep-learning approaches that target either attenuation correction or PET motion in isolation, OncoFocus provides a comprehensive, hardware-free solution that integrates correction for PET motion blur, attenuation-correction mismatch, and PET–CT misregistration within a single end-to-end workflow.

This study has several limitations. First, although breath-hold acquisition was initially considered a reference standard, it was ultimately not implemented due to inconsistent patient compliance and compromised image quality under breath-hold conditions. Future investigations with optimized acquisition conditions and protocols may obtain a more reliable reference standard for RMC validation. Second, several physiologic and psychological factors (such as respiratory pattern variability, anxiety, and fatigue) that may influence repeatability were not explicitly assessed. Future research in these areas may be helpful. Finally, our results of this study are based on a single PET/CT system, completed within a short recruitment period, with a limited sample size and without external validation. Multicenter studies with larger, more diverse patient populations, a broader range of diseases, and a longer follow-up period would help to validate and generalize our findings.

## 5. Conclusions

Respiratory-motion-corrected PET/CT demonstrated superior image quality, test–retest stability, and quantitative reproducibility compared with non-corrected reconstructions in patients with lung cancer. These findings support the routine clinical adoption of RMC, especially in cases where respiratory motion artifacts may affect lesion assessment or diagnostic confidence.

## Figures and Tables

**Table 1:** Stability analysis of RMC versus NMC in lesion measurements.

		ΔRMC vs. ΔNMC	ΔRMC-CB vs. ΔNMC-CB
SUV_max_	all lesion	(0.57 ± 0.77 vs. 0.88 ± 0.84) *	(0.63 ± 0.58 vs. 3.19 ± 8.33) *
	MU-Lobe group	(0.56 ± 0.93 vs. 1.07 ± 0.99) *	(0.68 ± 0.66 vs. 1.34 ± 1.39) *
	L-Lobe group	(0.58 ± 0.55 vs. 0.85 ± 0.72) *	(0.58 ± 0.49 vs. 5.43 ± 12.17) *
SUV_mean_	all lesion	(0.40 ± 0.53 vs. 0.43 ± 0.44) ^ns^	(0.36 ± 0.35 vs. 0.77 ± 1.08) *
	MU-Lobe group	(0.44 ± 0.55 vs. 0.51 ± 0.53) ^ns^	(0.42 ± 0.41 vs. 0.61 ± 0.80) *
	L-Lobe group	(0.33 ± 0.53 vs. 0.33 ± 0.29) ^ns^	(0.29 ± 0.25 vs. 0.97 ± 1.63) *
Length of lesion	all lesion	(0.07 ± 0.10 vs. 0.67 ± 1.01) **	(0.17 ± 0.25 vs. 1.36 ± 0.90) **
	MU-Lobe group	(0.04 ± 0.02 vs. 0.41 ± 0.43) **	(0.18 ± 0.33 vs. 0.73 ± 0.81) **
	L-Lobe group	(0.03 ± 0.13 vs. 0.99 ± 1.13) **	(0.14 ± 0.24 vs. 2.31 ± 1.44) **
MTV	all lesion	(0.03 ± 0.03 vs. 0.83 ± 0.90) **	(0.03 ± 0.02 vs. 9.24 ± 26.79) **
	MU-Lobe group	(0.03 ± 0.03 vs. 0.73 ± 0.87) **	(0.03 ± 0.02 vs. 2.80 ± 7.65) **
	L-Lobe group	(0.03 ± 0.04 vs. 0.95 ± 0.94) **	(0.03 ± 0.02 vs. 17.79 ± 37.96) **
TLG	all lesion	(6.03 ± 27.61 vs. 8.30 ± 26.98) ^ns^	(5.34 ± 22.55 vs. 49.33 ± 187.98) *
	MU-Lobe group	(3.06 ± 6.73 vs. 3.10 ± 4.61) ^ns^	(4.40 ± 10.66 vs. 5.75 ± 11.20) *
	L-Lobe group	(9.23 ± 40.22 vs. 14.80 ± 38.89) *	(6.00 ± 31.57 vs. 108.50 ± 267.79) *

All data are presented in the form of mean ± standard deviation; * *p* < 0.05, ** *p* < 0.01, and ^ns^ not significant. ΔRMC = |(RMC of Scan1) − (RMC of Scan2)|; ΔNMC = |(NMC of Scan1) − (NMC of Scan2)|; ΔRMC-CB= |(RMC of Scan2) − (RMC of ScanCB)|; and ΔNMC-CB = |(NMC of Scan2) − (NMC of ScanCB)|. MU-Lobe: middle-upper lobe; L-Lobe: lower lobe; MTV: metabolic tumor volume; and TLG: total lesion glycolysis.

**Table 2:** The CV of RMC versus NMC in lesion measurements.

		RMC Group	NMC Group	T	*p*
SUV_max_	All lesion	7.51 ± 6.67	22.68 ± 27.47	−3.039	0.005
	MU-Lobe group	6.83 ± 6.12	15.82 ± 6.95	−5.046	<0.001
	L-Lobe group	8.34 ± 7.42 ^ns^	31.01 ± 39.31 *	−2.118	0.054
SUV_mean_	All lesion	8.32 ± 6.26	16.19 ± 13.11	−4.141	<0.001
	MU-Lobe group	8.21 ± 5.57	13.65 ± 6.63	−4.282	<0.001
	L-Lobe group	8.44 ± 7.24 ^ns^	19.27 ± 18.0 *	−2.812	0.015
Length of lesion	All lesion	4.76 ± 5.29	38.26 ± 22.29	−8.660	<0.001
	MU-Lobe group	4.82 ± 4.99	44.54 ± 22.80	−6.930	<0.001
	L-Lobe group	4.68 ± 5.82 ^ns^	48.86 ± 25.31 *	−5.181	<0.001
MTV	All lesion	1.03 ± 1.01	25.93 ± 31.14	−4.510	<0.001
	MU-Lobe group	1.03 ± 1.02	19.41 ± 15.55	−4.951	<0.001
	L-Lobe group	1.03 ± 1.02 ^ns^	33.83 ± 42.64 ^ns^	−2.918	0.012
TLG	All lesion	8.28 ± 6.02	20.81 ± 30.38	−2.409	0.022
	MU-Lobe group	8.39 ± 5.65	13.68 ± 9.76	−2.135	0.049
	L-Lobe group	8.15 ± 6.66 ^ns^	29.46 ± 43.19 ^#^	−1.961	0.072

All data are presented in the form of mean ± standard deviation, and the superscript indicates the statistical difference between the MU-Lobe group and L-Lobe group; ^#^ *p* < 0.1, * *p* < 0.05, and ^ns^ not significant. CV: coefficients of variation; MU-Lobe: middle-upper lobe; L-Lobe: lower lobe; MTV: metabolic tumor volume; TLG: total lesion glycolysis.

**Table 3:** The ICC of lesion measurements in both RMC and NMC groups.

		SUV_max_	SUV_mean_	Length of Lesion	MTV	TLG
RMC group	All lesion	0.984 **	0.984 **	0.997 **	0.995 **	0.996 **
	MU-Lobe group	0.986 **	0.998 **	0.995 **	0.999 **	0.999 **
	L-Lobe group	0.971 **	0.968 **	0.997 **	0.995 **	0.995 **
NMC group	All lesion	0.505 **	0.908 **	0.846 **	0.976 **	0.969 **
	MU-Lobe group	0.957 **	0.956 **	0.883 **	0.999 **	0.999 **
	L-Lobe group	0.196 ^ns^	0.729 **	0.624 **	0.972 **	0.967 **

** *p* < 0.01, and ^ns^ not significant. ICC: intraclass correlation coefficient, MU-Lobe: middle-upper lobe; L-Lobe: lower lobe; MTV: metabolic tumor volume; TLG: total lesion glycolysis.

## Data Availability

The data that support the findings of this study are available from the corresponding author upon reasonable request.

## References

[1] Lillington J., Brusaferri L., Kläser K., Shmueli K., Neji R., Hutton B.F., Fraioli F., Arridge S., Cardoso M.J., Ourselin S. (2020). PET/MRI attenuation estimation in the lung: A review of past, present, and potential techniques. Med. Phys..

[2] Anan N., Zainon R., Tamal M. (2022). A review on advances in (18)F-FDG PET/CT radiomics standardisation and application in lung disease management. Insights Imaging.

[3] Dahlsgaard-Wallenius S.E., Hildebrandt M.G., Johansen A., Vilstrup M.H., Petersen H., Gerke O., Høilund-Carlsen P.F., Morsing A., Andersen T.L. (2021). Hybrid PET/MRI in non-small cell lung cancer (NSCLC) and lung nodules-a literature review. Eur. J. Nucl. Med. Mol. Imaging.

[4] Lamare F., Bousse A., Thielemans K., Liu C., Merlin T., Fayad H., Visvikis D. (2022). PET respiratory motion correction: Quo vadis?. Phys. Med. Biol..

[5] Ippoliti M., Lukas M., Brenner W., Schatka I., Furth C., Schaeffter T., Makowski M.R., Kolbitsch C. (2021). Respiratory motion correction for enhanced quantification of hepatic lesions in simultaneous PET and DCE-MR imaging. Phys. Med. Biol..

[6] Pépin A., Daouk J., Bailly P., Hapdey S., Meyer M.E. (2014). Management of respiratory motion in PET/computed tomography: The state of the art. Nucl. Med. Commun..

[7] Schlarbaum K.E. (2024). PET/CT Imaging in Lung Cancer. J. Nucl. Med. Technol..

[8] Manafi-Farid R., Askari E., Shiri I., Pirich C., Asadi M., Khateri M., Zaidi H., Beheshti M. (2022). [(18)F]FDG-PET/CT Radiomics and Artificial Intelligence in Lung Cancer: Technical Aspects and Potential Clinical Applications. Semin. Nucl. Med..

[9] Lee H.J., Son H.J., Yun M., Moon J.W., Kim Y.N., Woo J.Y., Lee S.H. (2021). Prone position [(18)F]FDG PET/CT to reduce respiratory motion artefacts in the evaluation of lung nodules. Eur. Radiol..

[10] Cheng Z., Chen L., Wang X., Wang Y., Zhao M., Zan K., Liu W., Cui X., Chai L., Ge M. (2023). Role of breath-hold lung PET in stage IA pulmonary adenocarcinoma. Insights Imaging.

[11] Pan T., Thomas M.A., Luo D. (2022). Data-driven gated CT: An automated respiratory gating method to enable data-driven gated PET/CT. Med. Phys..

[12] Feng T., Wang J., Sun Y., Zhu W., Dong Y., Li H. (2018). Self-Gating: An Adaptive Center-of-Mass Approach for Respiratory Gating in PET. IEEE Trans. Med. Imaging.

[13] Lu Y., Kang F., Zhang D., Li Y., Liu H., Sun C., Zeng H., Shi L., Zhao Y., Wang J. (2024). Deep learning-aided respiratory motion compensation in PET/CT: Addressing motion induced resolution loss, attenuation correction artifacts and PET-CT misalignment. Eur. J. Nucl. Med. Mol. Imaging.

[14] Shan Y., Yan S.Z., Wang Z., Cui B.X., Yang H.W., Yuan J.M., Yin Y.-Y., Shi F., Lu J. (2023). Impact of brain segmentation methods on regional metabolism quantification in (18)F-FDG PET/MR analysis. EJNMMI Res..

[15] Gratz M., Ruhlmann V., Umutlu L., Fenchel M., Hong I., Quick H.H. (2020). Impact of respiratory motion correction on lesion visibility and quantification in thoracic PET/MR imaging. PLoS ONE.

[16] Guerra L., Ponti E., Morzenti S., Spadavecchia C., Crivellaro C. (2017). Respiratory Motion Management in PET/CT: Applications and Clinical Usefulness. Curr. Radiopharm..

[17] Dayarathna S., Islam K.T., Uribe S., Yang G., Hayat M., Chen Z. (2024). Deep learning based synthesis of MRI, CT and PET: Review and analysis. Med. Image Anal..

[18] Hashimoto F., Onishi Y., Ote K., Tashima H., Reader A.J., Yamaya T. (2024). Deep learning-based PET image denoising and reconstruction: A review. Radiol. Phys. Technol..

[19] Chen X., Liu C. (2023). Deep-learning-based methods of attenuation correction for SPECT and PET. J. Nucl. Cardiol..

[20] Meng Q.L., Yang R., Wu R.Z., Xu L., Liu H., Yang G., Dong Y., Wang F., Chen Z., Jiang H. (2023). Evaluation of a respiratory motion-corrected image reconstruction algorithm in 2-[(18)F]FDG and [(68)Ga]Ga-DOTA-NOC PET/CT: Impacts on image quality and tumor quantification. Quant. Imaging Med. Surg..

[21] Koopman D., Jager P.L., Slump C.H., Knollema S., van Dalen J.A. (2019). SUV variability in EARL-accredited conventional and digital PET. EJNMMI Res..

[22] Deller T.W., Khalighi M.M., Jansen F.P., Glover G.H. (2018). PET Imaging Stability Measurements During Simultaneous Pulsing of Aggressive MR Sequences on the SIGNA PET/MR System. J. Nucl. Med..

[23] Al-Nabhani K.Z., Syed R., Michopoulou S., Alkalbani J., Afaq A., Panagiotidis E., O’mEara C., Groves A., Ell P., Bomanji J. (2014). Qualitative and quantitative comparison of PET/CT and PET/MR imaging in clinical practice. J. Nucl. Med..

[24] Kajiyama A., Ito K., Watanabe H., Mizumura S., Watanabe S.I., Yatabe Y., Gomi T., Kusumoto M. (2022). Consistency and prognostic value of preoperative staging and postoperative pathological staging using (18)F-FDG PET/MRI in patients with non-small cell lung cancer. Ann. Nucl. Med..

[25] Farolfi A., Calderoni L., Mattana F., Mei R., Telo S., Fanti S., Castellucci P. (2021). Current and Emerging Clinical Applications of PSMA PET Diagnostic Imaging for Prostate Cancer. J. Nucl. Med..

